# Effectiveness of ivermectin mass drug administration in the control of soil-transmitted helminth infections in endemic populations: a systematic review and meta-analysis

**DOI:** 10.1186/s40249-024-01185-5

**Published:** 2024-02-18

**Authors:** Brandon Le, Naomi E. Clarke, Nicolas Legrand, Susana Vaz Nery

**Affiliations:** The Kirby Institute, University of New South Wales, Level 6, Wallace Wurth Building, Sydney, NSW 2052 Australia

**Keywords:** Soil-transmitted helminths, Ivermectin, Albendazole, Mass drug administration, Preventive chemotherapy

## Abstract

**Background:**

Current soil-transmitted helminth (STH) control guidelines endorse the use of albendazole or mebendazole for school-based targeted preventive chemotherapy (PC), yet their reduced efficacy against *Strongyloides stercoralis* and *Trichuris trichiura* presents significant limitations. Emerging evidence indicates that community-wide PC [or mass drug administration (MDA)] using ivermectin, commonly used in other neglected tropical disease (NTD) control programs, may play an important role in controlling these parasites. We conducted a systematic review and meta-analysis to evaluate the effectiveness of ivermectin PC in reducing STH prevalence in endemic populations.

**Methods:**

We searched Pubmed, EMBASE, and Web of Science on February 14, 2023, for studies that investigated the effectiveness of ivermectin PC, either alone or in combination with other anthelmintic drugs, on STH infections, and provided a measure of STH prevalence before and after PC. We calculated pooled prevalence reductions for each STH using random-effects meta-analyses. Our protocol is available on PROSPERO (registration number CRD42023401219).

**Results:**

A total of 21 were eligible for the systematic review, of which 15 were eligible for meta-analysis. All studies delivered ivermectin through MDA. The pooled prevalence reduction of *S. stercoralis* following MDA with ivermectin alone was 84.49% (95% *CI* 54.96–94.66) across five studies and 81.37% (95% *CI* 61.62–90.96) across seven studies with or without albendazole. The prevalence reduction of *T. trichiura* was 49.93% (95% *CI* 18.23–69.34) across five studies with ivermectin alone, and 89.40% (95% *CI* 73.66–95.73) across three studies with the addition of albendazole. There was high heterogeneity for all syntheses (*I*^2^ > 65%).

**Conclusions:**

This study underscores the key role of ivermectin-based MDA in addressing limitations in current global STH guidelines in terms of limited efficacy against *S. stercoralis* and *T. trichiura*. Based on these findings, revising international STH guidelines to include ivermectin is a promising option to progress the control and eventual elimination of STHs and other NTDs.

**Supplementary Information:**

The online version contains supplementary material available at 10.1186/s40249-024-01185-5.

## Background

Soil-transmitted helminth (STH) infections are the most prevalent neglected tropical disease (NTD) worldwide, infecting an estimated 895 million people [1] and contributing 1.9 million disability-adjusted life years (DALYs) per annum [2]. STH infections are caused by a group of intestinal nematodes including *Ascaris lumbricoides*, *Trichuris trichiura*, hookworms (*Necator americanus*, *Ancylostoma ceylanicum*, *Ancylostoma duodenale*) and *Strongyloides stercoralis*. Of note, *S. stercoralis* is estimated to infect 600 million people annually [3], and is characterised by its unique auto-infective lifecycle capable of causing chronic and potentially fatal hyperinfection among immunosuppressed patients [4]. *T. trichiura* also contributes significantly to the global burden with an estimated 290 million annual infections, and is associated with a range of adverse health outcomes in the case of chronic and heavy intensity infections, including developmental delay, anaemia, gastrointestinal disease, and *Trichuris* dysentery syndrome [1].

The mainstay of public health control efforts against STH infections is school-based targeted preventive chemotherapy (PC). PC refers to the large-scale distribution of safe and efficacious drugs to specific risk groups (targeted PC) or to entire communities [mass drug administration (MDA)]. For STHs, the WHO recommended strategy is school based targeted PC, delivering benzimidazole anthelmintics, mebendazole or albendazole, to school-aged children [5]. While other risk groups have been identified, namely women of reproductive age and adults in high-risk occupations like miners, there are no defined distribution channels to target them. Furthermore, although these drugs are efficacious against *A. lumbricoides* and hookworm [6], they have no efficacy against *S. stercoralis* [7] and limited efficacy against *T. trichiura* [6]. Concerns have also been raised regarding the potential generation of anthelmintic resistance among humans [8], a phenomenon well documented in livestock [9]. Investigation into alternative therapeutic regimens for STH control is therefore a key priority.

Ivermectin is a broad-spectrum antiparasitic drug that has high efficacy against *S. stercoralis* and *A. lumbricoides*, moderate efficacy against *T. trichiura*, and poor efficacy against hookworms [6, 10–13]. While ivermectin alone has not been used in school-based targeted PC programs for STH, it has been used over the last two decades in highly effective community-wide MDA campaigns against onchocerciasis [14] and more recently against scabies [15, 16]. These programs demonstrated, to varying degrees, reductions in the prevalence and intensity of off-target STHs [17–19], providing preliminary evidence that ivermectin MDA may be a cost-effective strategy for the simultaneous control of multiple NTDs, including STH infections. While the combination of albendazole and ivermectin has significant therapeutic efficacy against all STH species [6, 7], their effectiveness at the population level in MDA programs has also been variable [20–25].

Addressing these gaps in knowledge will provide an important evidence base to inform policy decision-making and optimise the implementation of PC programs to progress STH control and elimination targets. This systematic review and meta-analysis aimed to evaluate the role of ivermectin-based PC in improving the control of STHs by describing the existing literature that documents the impact of ivermectin PC in endemic populations, and to quantify its effectiveness, both as a standalone regimen and in combination with albendazole, in reducing STH prevalence.

## Methods

### Search strategy and selection criteria

This systematic review and meta-analysis was completed in accordance with the 2020 PRSIMA guidelines [26]. Papers were eligible for inclusion if they investigated the effectiveness of ivermectin PC (including both targeted PC and MDA), either alone or in combination with other anthelmintic drugs, on STH infections, and provided a measure of STH prevalence before and after PC.

Papers were excluded from the systematic review if PC was given only to positive cases or given only to a random sample of the population; if outcomes were only reported for positive cases; if PC was delivered in the context of randomised trials where randomisation occurred at individual level; or if data published were duplicate data from another paper.

Additional exclusion criteria were applied for the quantitative synthesis (meta-analysis) to remove significant sources of heterogeneity. Studies were excluded from the meta-analysis if they met any of the following three exclusion criteria: (1) time from the last round of ivermectin PC to the follow-up prevalence assessment was less than 1 month or greater than 24 months as studies with < 1 month follow-up periods will likely be measuring PC efficacy (intervention outcomes under ideal settings) rather than effectiveness (intervention outcomes under real-world settings), therefore overestimating the real-world impact of PC, while those with > 24 months follow-up will likely underestimate the effectiveness of PC; (2) if the time from the baseline assessment to the first round of PC was greater than 12 months; or (3) if the baseline prevalence was less than 5%.

We searched Pubmed, EMBASE, and Web of Science on February 14, 2023 with no limitations on year or language of publication. The following search terms were used for each of the three key concepts: (1) ivermectin: “ivermectin”; (2) PC: “preventive chemotherapy” or “chemotherapy” or “mass drug administration” or “mass administration” or “mass treatment” or “population” or “community” or “communities” or “village” or “villages” or “school” or “schools” or “prevalence” or “program” or “programme”; (3) soil-transmitted helminth infections: “soil-transmitted helminth” or “soil-transmitted helminths” or “STH” or “nematode” or “geohelminth” or “*Ascaris*” or “hookworm” or “*Necator*” or “*Ancylostoma*” or “*Trichuris*” or “*Strongyloides*”. The complete search strategy, including medical subject heading terms used, are provided in Additional file 1 (p. 1). We identified additional studies by manually searching reference lists of included papers and key systematic reviews [27, 28], and through personal knowledge.

All papers retrieved from databases were imported into EndNote version X9 (Clarivate, Philadelphia, USA) where they were first deduplicated. All paper titles, abstracts, and full-text papers were then screened by BL. For quality control, 20% of the papers subjected to full text screening were randomly selected and independently reviewed by NL to determine discrepancies in inclusion or exclusion. All papers were assessed for eligibility against the review protocol, available in PROSPERO (registration number CRD42023401219).

### Data extraction and quality assessment

Data were extracted by BL using Covidence [29]. Where available, the following data were extracted from eligible papers: year and country of study; study population; drug(s) used; diagnostic technique; study design; primary disease target of PC; strategy of drug administration (mass or targeted); drug dosage and frequency; number of rounds of drug distribution; intervention coverage; additional interventions employed for STH control; prevalence of infection for each species before and after PC (including number of participants receiving treatment, number of participants infected, and proportion infected); time between baseline and each of the follow-up prevalence assessments; and follow-up time from last round (time between the last round of PC and follow-up prevalence assessments). For quality control, 20% of the papers subjected to data extraction were randomly selected and independently reviewed by NL, where any discrepancies were resolved through discussion. In the case of disagreements, it was escalated to NEC who made the final decision.

If a paper reported data for multiple arms, we extracted data from all arms separately provided they represented independent populations, herein referred to as “studies”. If a range of intervention coverage was reported, we extracted the median. In trials with a control group that was provided PC only and an intervention group that received an additional intervention [e.g., water, sanitation, and hygiene (WASH) improvements], only data from the control groups were extracted. Where multiple follow-up prevalence assessments were reported, we extracted data for all assessments.

We contacted five authors for additional data requesting a full manuscript or published paper for conference abstracts (*n* = 3) and intervention coverage data (*n* = 2). Three authors responded, of which one was unable to share a manuscript documenting the results of a conference abstract and two were unable to share coverage data.

Study quality was assessed using an approach from a previously published systematic review [27] that used a modified scale adapted from a validated scale [30] designed to assess risk of bias in prevalence studies. Modifications were made to account for most studies being quasi-experimental, pre-post prevalence surveys without a control group. We used quality assessment tools from the National Heart, Lung, and Blood Institute for observational and pre-post study designs [31, 32], and made modifications to ensure consistent participant selection and intervention coverage [27]. We evaluated studies based on nine safeguards to eliminate bias in measuring STH prevalence, including items that evaluated internal and external validity. Quality assessment was performed by BL and independently reviewed by NEC, with disagreements resolved through consensus.

### Statistical analysis

All analyses were completed separately for each STH species and for each PC regimen. In the primary analysis, we conducted a random-effects weighted meta-analysis evaluating the effectiveness of PC against each STH species. The first timepoint at which prevalence data were available was considered the baseline (p_1_). For the purpose of the meta-analysis, we used one follow-up prevalence estimate (p_2_) per study, defined as the prevalence assessment that followed the last round of PC involving ivermectin. Although the primary outcome in the model was the pooled prevalence ratio (p_2_/p_1_), we converted and reported these results as pooled prevalence reduction [(1 − pooled prevalence ratio) × 100] for ease of interpretation. We report the pooled prevalence reduction for ivermectin PC as a monotherapy regimen and, separately, ivermectin and albendazole PC for *A. lumbricoides*, *T. trichiura*, and hookworm. Given that a single dose of albendazole is unlikely to have significant therapeutic activity against *S. stercoralis* [10], we pooled all studies using ivermectin for this species. Where sufficient data were available (*n* > 2 studies), we conducted sensitivity analyses based on the following restrictions: (1) follow-up time from last round (> 6 months; > 6 months and ≤ 18 months); (2) number of PC rounds administered (one round vs. multiple rounds); (3) diagnostic technique (stool-based methods for *S. stercoralis*; Kato-Katz for *A. lumbricoides*, *T. trichiura*, and hookworm); (4) PC coverage (< 75% vs ≥ 75% as recommended by the WHO for STH control); and (5) studies using ivermectin alone for *S. stercoralis*. Heterogeneity was assessed using Cochran’s Q test and Higgins’* I*^2^ where an *I*^2^ of greater than 50% was considered to indicate significant heterogeneity. Publication bias and evidence of small-study effects were assessed through visual inspection of Doi plots and the Luis Furuya-Kanamori (LFK) index, which have greater sensitivity and power than the funnel plot and Egger’s regression method when there are fewer studies [33]. Visual asymmetry on the Doi plot refers to an imbalanced distribution of effect sizes, indicating potential publication bias where small or non-significant effect sizes may be missing from the analysis. An LFK index of ± 1 indicates no asymmetry on the Doi plot, between ± 1 and ± 2 indicates minor symmetry, and ± 2 indicates major asymmetry [33].

In the secondary analysis we used random-effects weighted meta-regressions to quantify the effect of select covariates on PC effectiveness. This was due to the presence of heterogeneity across studies on variables likely to affect PC effectiveness, including baseline prevalence, number of rounds of PC, and follow-up time from last round. For the purposes of the meta-regression, we used data from all follow-up prevalence assessments if a study had multiple assessments, where prevalence reduction was defined as the relative difference between each follow-up assessment (p_2,_ or p_3,_ or p_4_ …) and the first timepoint at which data were available (p_1_), yielding multiple reduction estimates for these studies [(1 − p_2_/p_1_) × 100) and; (1 − p_3_/p_1_) × 100) and; (1 − p_4_/p_1_) × 100) …]. The outcome variable in the model was relative prevalence reduction, where it was winsorised at its lower boundary so that any prevalence increase was reset to zero, allowing the winsorised distribution to mirror that of a proportion and to be analysed with a logit link function [27]. This approach was considered appropriate as any increase in prevalence would be unrelated to PC, suggesting no effectiveness. We entered the following covariates in the model: (1) baseline prevalence (%), (2) number of rounds of PC between baseline and follow-up prevalence assessments, and (3) time from last round of PC to follow-up prevalence assessments (months). We obtained the weighted odds ratio by exponentiating the coefficients, and report this estimate with the associated 95% confidence intervals (*CI*s).

All meta-regression models were fitted with robust clustered standard errors to account for clustering at the study level and heteroscedasticity. We only had sufficient data to analyse the impact of these covariates on the effectiveness of ivermectin PC, with or without albendazole, against *S. stercoralis*, and ivermectin and albendazole against *A. lumbricoides*, *T. trichiura*, and hookworm. Meta-analyses were completed using MetaXL (version 5.3) and meta-regressions were completed using Stata (version 17.0).

## Results

### Systematic review

**Fig. 1:**
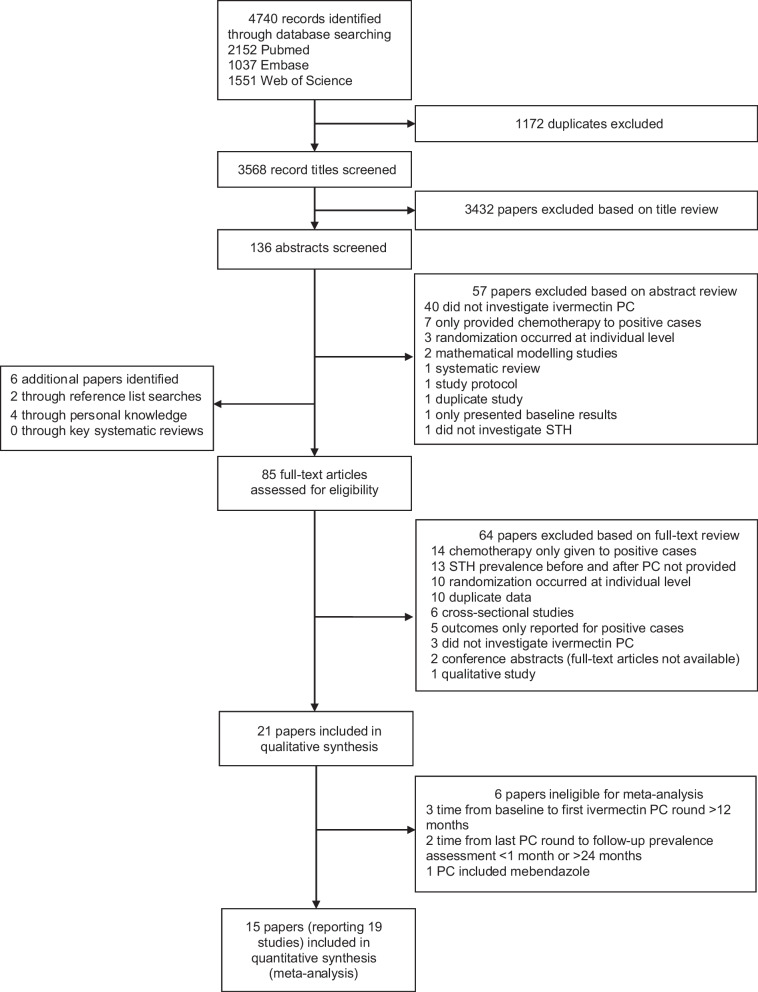
Study selection flowchart

A total of 21 papers (reporting 25 studies) met the inclusion criteria for the systematic review [17–25, 34–45] and 15 of these papers (reporting 19 studies) were included in the meta-analysis [17–22, 24, 25, 35, 36, 39, 41–43, 45] (Fig. 1). Papers excluded at the full-text screening stage are provided in Additional file 1 (p. 2–4).

Table 1 lists individual studies included in the systematic review with their characteristics while Table 2 provides an aggregated summary of the study characteristics. Of the 25 studies included in the systematic review, 17 (68%) used ivermectin and albendazole as their PC regimen [20–25, 34, 36, 38–41, 43, 45] and 8 (32%) used ivermectin with no other anthelminthic drugs [17–19, 35, 37, 42, 44]. Nine studies used additional drugs in addition to ivermectin and/or albendazole [19, 34, 36, 38–41, 44]. There was no head-to-head comparison of ivermectin versus ivermectin and albendazole. Ivermectin was distributed to entire through community-wide MDA for all studies [17–25, 34–45]. Onchocerciasis (*n* = 3) (17, 34, 37) and scabies (*n* = 3) (18, 39, 43) were the primary diseases targeted for studies using ivermectin only. In studies using ivermectin and albendazole combined, albendazole was mostly distributed to entire communities through MDA together with ivermectin (*n* = 13) [21–25, 36, 39, 41, 43, 45], although four studies complemented MDA efforts with school-based targeted PC [20, 34, 38, 40] with albendazole only on alternating timelines. Lymphatic filariasis was the most commonly targeted primary disease, representing 64% of these studies (*n* = 16) (19, 21, 22, 33, 35, 36, 38, 40–42, 44, 46). Ivermectin dosages were within standard range (one dose, 150–200 μg/kg) for most studies (*n* = 18, 72%) where dosage was reported [17–22, 24, 25, 34–37, 40–44]. Two studies administered two doses given 7 to 14 days apart for scabies [17, 42] while one study [44] used a higher dosage (400 μg/kg) for lymphatic filariasis.

**Table 1:** Studies included in systematic review

Authors and year	Study population	Study location	STH studied^b^	Diagnostic technique	Primary disease target(s) of chemotherapy	PC regimen	Administration strategy	*N* rounds^c^	Ivermectin dosage	Albendazole dosage	Follow-up time after last round	PC coverage
Ame et al. 2022	Children	Tanzania	A, T, H	Kato Katz	Lymphatic filariasis	Ivermectin, albendazole, praziquantel	ALB PC to SAC + ALB + IVM MDA + ALB + PZQ MDA	3	Single dose (200 μg/kg)	Single dose (400 mg)	36 months	82%
Anselmi et al. 2015^a^	Adults	Ecuador	A, T, H, S	Microscopy (direct stool examination and formol-ether-concentration)	Onchocerciasis	Ivermectin	IVM MDA	5	Single dose (150 μg/kg)	Not applicable	1 month	89%
Bah et al. 2019^a^	Children	Sierra Leone	A, T, H	Kato Katz	Lymphatic filariasis, STH	Ivermectin, albendazole	ALB PC to SAC + ALB + IVM MDA	5	Single dose (200 μg/kg)	Single dose (400 mg)	8 months	75%
Echazu et al. 2017^a^	Community	Argentina	H, S	Five techniques: (1) Microscopy (sedimentation method) (2) Agar plate culture (3) Harada-Mori filter-paper culture (4) Baermann concentration (5) McMaster method	STH	Ivermectin, albendazole	ALB + IVM MDA	2	Single dose (200 μg/kg)	Single dose (400 mg)	17 months	58%
Eneanya et al. 2021 arm 1^a^	Community	Liberia	A, T, H	Kato Katz	Lymphatic filariasis	Ivermectin, albendazole	ALB + IVM MDA (annually)	3	Single dose (200 μg/kg)	Single dose (400 mg)	12 months	27%
Eneanya et al. 2021 arm 2^a^	Community	Liberia	A, T, H	Kato Katz	Lymphatic filariasis	Ivermectin, albendazole	ALB + IVM MDA (semi-annually)	5	Single dose (200 μg/kg)	Single dose (400 mg)	12 months	35%
Eneanya et al. 2022 arm 1^a^	Community	Liberia	A, T, H	Kato Katz	Lymphatic filariasis, onchocerciasis, schistosomiasis, STH	Ivermectin, albendazole, praziquantel	ALB + IVM + PZQ MDA (annually)	4	Single dose (200 μg/kg)	Single dose (400 mg)	24 months	56%
Eneanya et al. 2022 arm 2^a^	Community	Liberia	A, T, H	Kato Katz	Lymphatic filariasis, onchocerciasis, schistosomiasis, STH	Ivermectin, albendazole, praziquantel	ALB + IVM + PZQ MDA (semi-annually)	7	Single dose (200 μg/kg)	Single dose (400 mg)	24 months	53%
Gebrezgabiher et al. 2022	Community	Ethiopia	A, T, H	Microscopy (Ritchie-Frick technique and Kato Katz)	Onchocerciasis	Ivermectin	IVM MDA	3	Single dose (150 μg/kg)	Not applicable	7 months	88%
Griswold et al. 2022	Children	Nigeria	A, T, H	Kato Katz	Schistosomiasis, STH	Ivermectin, albendazole, mebendazole, praziquantel	IVM MDA IVM + ALB MDA PZQ MDA ALB PC to SAC MEB PC to SAC	5	Not reported	Not reported	10 months	76%
Heukelbach et al. 2004^a^	Community	Brazil	A, T, H	Microscopy (sedimentation method)	Parasitic skin diseases	Ivermectin	IVM MDA	1	Two doses (200 μg/kg), 10 days apart	Not applicable	9 months	83%
Hurlimann et al. 2018^a^	Community	Cote d'Ivoire	A, T, H	Kato Katz	Lymphatic filariasis, onchocerciasis, schistosomiasis, STH	Ivermectin, albendazole, praziquantel	IVM + ALB + PZQ MDA	2	Not reported	Not reported	5 months	Not reported
Knopp et al. 2009	Children	Zanzibar	A, T, H, S	Kato Katz for *A. lumbricoides*, *T. trichiura*, hookworm Baermann for *S. stercoralis*	Lymphatic filariasis	Ivermectin, albendazole, mebendazole, praziquantel	IVM + ALB MDA ALB + PZQ PC to SAC MEB + PZQ PC to SAC	16	Single dose (200 μg/kg)	Single dose (400 mg)	7 months	Not reported
Knudson et al. 2012^a^	Community	Colombia	A, T, S	Microscopy (Ritchie-Frick technique)	Onchocerciasis	Ivermectin	IVM MDA	23	Single dose (200 μg/kg)	Not applicable	4 months	86%
Le et al. 2023b^a^	Children	Timor-Leste	A, T, H, S	Quantitative PCR	Lymphatic filariasis, STH	Ivermectin, diethylcarbamazine citrate, albendazole	IDA MDA	1	Single dose (200 μg/kg)	Single dose (400 mg)	18 months	76%
Le et al. 2023a arm 1^a^	Community	Solomon Islands	A, T, H, S	Quantitative PCR	Scabies	Ivermectin	IVM MDA (1 dose)	1	Single dose (200 μg/kg)	Not applicable	21 months	93%
Le et al. 2023a arm 2^a^	Community	Solomon Islands	A, T, H, S	Quantitative PCR	Scabies	Ivermectin	IVM MDA (2 doses)	1	Two doses (150 to 200 μg/kg), 7 to 14 days apart	Not applicable	21 months	81%
Loukouri et al. 2020 arm 1^a^	Community	Cote d'Ivoire	H	Kato Katz	Lymphatic filariasis	Ivermectin, albendazole	ALB + IVM MDA (annually)	3	Single dose (200 μg/kg)	Single dose (400 mg)	12 months	65%
Loukouri et al. 2020 arm 2^a^	Community	Cote d'Ivoire	H	Kato Katz	Lymphatic filariasis	Ivermectin, albendazole	ALB + IVM MDA (semi-annually)	5	Single dose (200 μg/kg)	Single dose (400 mg)	6 months	64%
Marks et al. 2020^a^	Children	Solomon Islands	S	Serology (bead-based immunoassay testing for antibodies against the recombinant NIE-antigen)	Scabies	Ivermectin, azithromycin	IVM + AZN MDA	1	Single dose (200 μg/kg)	Not applicable	12 months	91%
Massa et al. 2009^a^	Children	Tanzania	A, T, H	Kato Katz	Lymphatic filariasis, Schistosomiasis, STH	Ivermectin, albendazole	ALB + IVM MDA	1	Single dose (150 μg/kg)	Single dose (400 mg)	7 months	81%
Moulia-Pelat et al. 1995	Children	French Polynesia	A, T	Kato Katz	Lymphatic filariasis	Ivermectin, diethylcarbamazine citrate	ID MDA	1	Single dose (400 μg/kg)	Not applicable	1 week	Not reported
Vargas et al. 2017^a^	Community	Argentina	A, T, H, S	Serology (NIE-ELISA)	STH	Ivermectin, albendazole	ALB + IVM MDA	1	Single dose (200 μg/kg)	Single dose (400 mg)	17 months	Not reported
Ziem et al. 2006a	Community	Ghana	H	Kato Katz	Lymphatic filariasis, oesophagostomiasis	Ivermectin, albendazole	ALB + IVM MDA	1	Not reported	Single dose (400 mg)	6 months	73%
Ziem et al. 2006b^a^	Community	Ghana	H	Kato Katz	Lymphatic filariasis, oesophagostomiasis	Ivermectin, albendazole	ALB + IVM MDA	1	Not reported	Single dose (400 mg)	18 months	75%

*ALB* albendazole, *AZN* azithromycin, *ID* ivermectin and diethylcarbamazine citrate, *IDA* ivermectin, diethylcarbamazine, and albendazole, *IVM* ivermectin; *MDA* mass drug administration, *MEB* mebendazole, *PC* preventive chemotherapy, *PZQ* praziquantel, *SAC* school-aged children, *STH* soil-transmitted helminths

^a^Study included in meta-analysis

^b^A: *A. lumbricoides*; T: *T. trichiura*; H: hookworm; S: *S. stercoralis*

^c^Number of preventive chemotherapy rounds between baseline and the prevalence assessment used in meta-analysis, which included ivermectin

**Table 2:** Summary of study characteristics

Total studies included in systematic review	Ivermectin	Ivermectin and albendazole	All
	*n* (%)	*n* (%)	*n* (%)
	8 (100)	17 (100)	25 (100)
Location			
Africa	1 (13)	14 (82)	15 (60)
South America	3 (38)	2 (12)	5 (20)
South-East Asia	0	1 (6)	1 (4)
Western Pacific	4 (50)	0	4 (16)
Study population^a^			
Children	2 (25)	6 (35)	8 (32)
Adults	1 (13)	0	1 (4)
Community	5 (63)	11 (65)	16 (64)
Diagnostic method			
Microscopy	5 (63)	15 (88)	20 (80)
Molecular	2 (25)	1 (6)	3 (12)
Serology	1 (13)	1 (6)	2 (8)
Primary disease target of preventive chemotherapy^b^			
Lymphatic filariasis	1 (13)	14 (82)	15 (60)
Oesophagostomiasis	0	2 (12)	2 (8)
Onchocerciasis	3 (38)	3 (18)	6 (24)
Parasitic skin diseases or scabies	4 (50)	0	4 (16)
Schistosomiasis	0	5 (29)	5 (20)
Soil-transmitted helminths	0	9 (53)	9 (36)
Additional drugs used in preventive chemotherapy			
Azithromycin	1 (13)	0	1 (4)
Diethylcarbamazine citrate	1 (13)	1 (6)	2 (8)
Mebendazole	0	1 (6)	1 (4)
Praziquantel	0	5 (29)	5 (20)
Ivermectin administration strategy			
School-based only	0	0	0
Mass drug administration only	8 (100)	17 (100)	25 (100)
Albendazole administration strategy			
School-based only	NA	0	0
Mass drug administration only	NA	13 (76)	13 (52)
Both	NA	4 (24)	4 (16)
Number of rounds			
1	5 (63)	5 (29)	10 (40)
2–5	2 (25)	10 (59)	12 (48)
> 5	1 (13)	2 (12)	3 (12)
Follow-up time from last round			
≤ 6 months	3 (38)	3 (18)	6 (24)
> 6–12 months	3 (38)	7 (42)	10 (40)
> 12–24 months	2 (25)	6 (35)	8 (32)
> 24 months	0	1 (6)	1 (12)
Intervention coverage			
< 75%	0	8 (47)	8 (32)
≥ 75%	7 (88)	6 (35)	13 (52)
Not reported	1 (13)	3 (18)	4 (16)

*NA* not applicable

^a^Population that was recruited for the prevalence surveys

^b^Number of studies may exceed total as some studies had multiple primary disease targets

Percentages may exceed 100% due to rounding

The number of rounds of MDA administered between baseline and last prevalence assessment ranged between 1 and 23 rounds, with 10 (40%) studies administering only 1 round [17, 19, 22–24, 41, 42, 44, 45]. The follow-up time from last round ranged between 1 week and 36 months, with 16 (64%) studies being 12 months or less [17–20, 22, 23, 25, 35, 37–40, 43, 44], 8 (32%) being between > 12 and ≤ 24 months [21, 24, 36, 41, 42, 45], and 1 (4%) being greater than 24 months [34]. Of the 17 studies that assessed ivermectin and albendazole, there were between 1 and 16 rounds administered, with a median of 3 rounds. Follow-up time ranged between 5 and 36 months, with a median of 12 months. Of the 8 studies that assessed ivermectin, there were between 1 and 23 rounds administered, with a median of 1 round. Follow-up time ranged between 1 week and 21 months, with a median of 10.5 months. There was considerable variability in MDA coverage. Of the 21 (84%) studies that reported coverage [17–23, 25, 34–38, 41–43, 45], the range was between 27 and 93%, with 13 studies [17–20, 22, 34, 35, 37, 38, 41, 42, 45] achieving ≥ 75% coverage as recommended by the WHO for STH control. For studies assessing ivermectin and albendazole, coverage ranged between 27 and 82%, with a median of 73%, while the coverage for ivermectin alone studies ranged between 81 and 91%, with a median of 88%. Additional information about the studies included in the review, including locations, surveyed populations, diagnostic methods, and primary disease targets are summarised in Table 1 (for individual studies) and 2 (aggregated summary).

### Risk of bias

Results of the risk of bias assessment is summarised in Additional file 1 (p. 5). Notably, of the 15 papers included in the meta-analysis, there were 13 (87%) that had response (or participation) rates of < 75% or showed a difference in relevant demographic characteristics between responders (participants) and non-responders (non-participants) (or not reported). Additionally, there were 6 (40%) papers where the drug distribution strategy was not clearly described and/or not delivered to at least 75% of the target population (or not reported). There was no indication of major risk of bias among the remaining 7 items.

### Meta-analysis

**Fig. 2:**
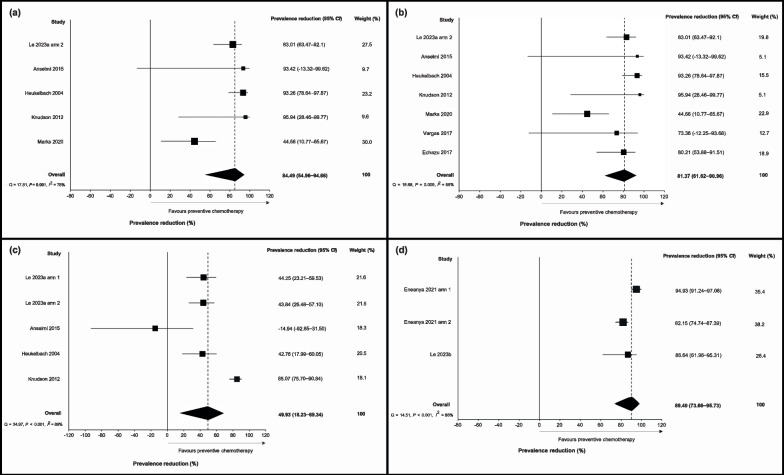
Forest plot of prevalence reduction for studies assessing the effectiveness of **a** ivermectin alone against *Strongyloides stercoralis*, **b** ivermectin, with or without albendazole, against *S. stercoralis*, **c** ivermectin alone against *Trichuris trichiura*, and **d** ivermectin and albendazole against *T. trichiura*

**Fig. 3:**
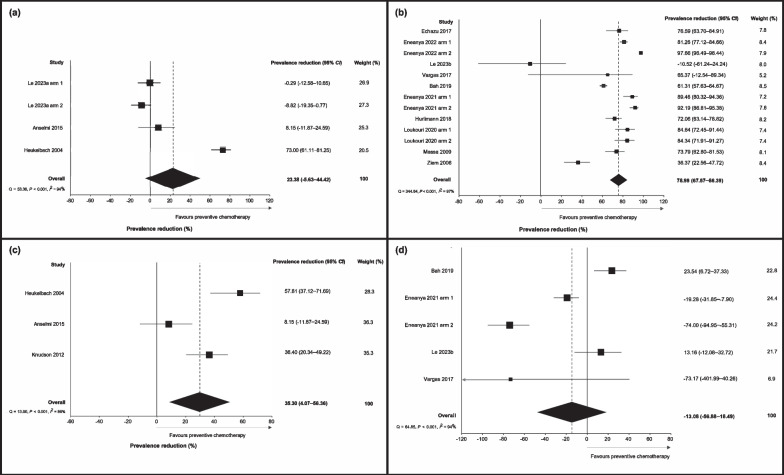
Forest plot of prevalence reduction for studies assessing the effectiveness of **a** ivermectin alone against hookworm **b** ivermectin and albendazole against hookworm, **c** ivermectin alone against *Ascaris lumbricoides*, and **d** ivermectin and albendazole against *A. lumbricoides*

Meta-analysis results are presented in Fig. 2 (for *S. stercoralis* and *T. trichiura*) and 3 (for hookworm and *A. lumbricoides*). The pooled prevalence reduction of *S. stercoralis* following MDA with ivermectin alone was 84.49% (95% *CI* 54.96–94.66) across five studies (Fig. 2a), and 81.37% (95% *CI* 61.62–90.96) across seven studies with or without albendazole (Fig. 2b). The pooled prevalence reduction for *T. trichiura* was 49.93% (95% *CI* 18.23–69.34) across five studies with ivermectin (Fig. 2c), and 89.40% (95% *CI* 73.66–95.73) across three studies with ivermectin and albendazole (Fig. 2d). We did not observe a significant reduction in hookworm prevalence with ivermectin alone [Fig. 3a, prevalence reduction 23.38% (95% *CI* − 5.63–44.42) across four studies], but the pooled prevalence reduction was 78.99% (95% *CI* 67.57–86.39) when using ivermectin and albendazole across 13 studies (Fig. 3b). Although we observed an *A. lumbricoides* prevalence reduction of 35.30% (95% *CI* 4.07–56.36) across three studies with ivermectin alone (Fig. 3c), we did not detect a statistically significant reduction associated with ivermectin and albendazole MDA across five studies [Fig. 3d, prevalence reduction − 13.08% (95% *CI* − 56.88–18.49)].

Results of the sensitivity analyses are in Additional file 1 (p. 8). Of note, the pooled prevalence reduction was sensitive to variations in the number of MDA rounds and coverage for hookworm. A single round of MDA resulted in a hookworm prevalence reduction of 47.14% (95% *CI* 0.95–71.79) across four studies, compared to 85.70% (95% *CI* 73.35–91.70) across nine studies where multiple rounds were administered. Reduced MDA coverage (< 75%) was associated with a hookworm prevalence reduction of 88.95% (95% *CI* 79.07–94.16) across 7 studies, compared with 48.80% (95% *CI* 19.73–67.34) associated with higher coverage (≥ 75%) across 4 studies. We did not observe variations in pooled prevalence reduction for the remaining sensitivity analyses or there was insufficient data (Additional file 1, p. 8).

There was high heterogeneity among all syntheses. For studies assessing ivermectin alone, *I*^2^ was 78% for *S. stercoralis,* 89% for *T. trichiura*; 94% for hookworm; 86% for *A. lumbricoides*, with a similar pattern when albendazole was considered (Figs. 2 and 3).

Results of Doi plots and LFK indices showed evidence of major asymmetry for studies assessing ivermectin alone for *S. stercoralis* (LFK index -7.35) and hookworm (LFK index − 3.71), but less for *A. lumbricoides* (LFK index − 1.24) and *T. trichiura* (LFK index − 0.36) (Additional file 1, p. 7). For studies assessing ivermectin and albendazole, there was major asymmetry for *S. stercoralis* (LFK index − 4.33), hookworm (LFK index − 3.21), and *T. trichiura* (LFK index − 2.58), with no evidence of asymmetry for *A. lumbricoides* (LFK index − 0.78) (Additional file 1, p. 6).

### Meta-regression

As shown in Table 3, for each 1% increase in baseline prevalence, there was significantly reduced odds of prevalence reduction for *T. trichiura* (*OR* = 0.95, 95% *CI* 0.90–0.99, *P* = 0.041) and *A. lumbricoides* (*OR* = 0.82, 95% *CI* 0.70–0.96, *P* = 0.027). For each increase in the number of MDA rounds, there was an 82% increase in the odds of prevalence reduction for hookworm (*OR* = 1.82, 95% *CI* 1.21–1.73, *P* = 0.008).

**Table 3:** Odds of prevalence reduction, stratified by STH

	Odds ratio (95% *CI*)	*P*	*R*^2^
*S. stercoralis* (*N* = 8)			
Baseline prevalence (%)	0.99 (0.80–1.23)	0.922	0.218
Number of chemotherapy rounds	1.16 (0.95–1.43)	0.119	
Follow-up time after last round (months)	1.06 (0.84–1.34)	0.571	
*T. trichiura* (*N* = 9)			
Baseline prevalence (%)	0.95 (0.90–0.99)	**0.041**	0.303
Number of chemotherapy rounds	1.35 (0.85–2.14)	0.109	
Follow-up time after last round (months)	0.94 (0.83–1.07)	0.171	
Hookworm (*N* = 30)			
Baseline prevalence (%)	1.01 (0.93–1.10)	0.759	0.257
Number of chemotherapy rounds	1.82 (1.21–2.73)	**0.008**	
Follow-up time after last round (months)	0.90 (0.70–1.18)	0.444	
*A. lumbricoides* (*N* = 11)			
Baseline prevalence (%)	0.82 (0.70–0.96)	**0.027**	0.748
Number of chemotherapy rounds	0.93 (0.36–2.40)	0.849	
Follow-up time after last round (months)	1.07 (0.68–1.66)	0.708	

Random effects weighted meta-regression with robust clustered standard errors

Embolden values indicate statistically significant results at the *P* < 0.05 level

*S. stercoralis* analysis included studies using ivermectin, with or without albendazole

*T. trichiura*, hookworm, and *A. lumbricoides* analyses included studies using ivermectin and albendazole

## Discussion

For the past three decades, WHO guidelines for STH control have endorsed specific therapeutic regimens that have been widely implemented in school-based targeted PC programs. However, these drugs have inadequate efficacy against *S. stercoralis* and *T. trichiura* and school-based targeted PC does not address adult reservoir of infections [46, 47]. This systematic review and meta-analysis is the first synthesis of existing evidence on the effectiveness of ivermectin PC, both as a standalone regimen and in combination with albendazole, in reducing STH prevalence in children and adults.

We observed a substantial reduction in *S. stercoralis* prevalence following ivermectin MDA. This is consistent with findings from a recent meta-analysis [28] and adds to the growing body of evidence supporting the inclusion of ivermectin in control guidelines for *S. stercoralis*. While the WHO has begun addressing gaps in current guidelines, a comprehensive strategy for *S. stercoralis* control is still under development. Our study highlights three important gaps in understanding that will need to be addressed to inform WHO recommendations. First, all studies delivered ivermectin through community-wide MDA, with impact observed in both children and adults, whereas current STH control is based on school-based targeted PC. Given that morbidity from *S. stercoralis* infections is concentrated in adults [47], guidelines may advocate for MDA. Second, the modest sample sizes and baseline prevalence of *S. stercoralis* infections (< 20%) means that it is unclear, based on the available evidence, how thresholds for MDA should be determined. Finally, there remain uncertainties regarding the added benefit of administering multiple rounds of MDA. Most existing studies assessed the impact of only 1 to 2 rounds, often with variable follow-up periods. Further research and mathematical modelling are needed to address these gaps.

While MDA with ivermectin alone resulted in a moderate reduction in *T. trichiura* prevalence, its co-administration with albendazole yielded a more substantial reduction, consistent with the efficacy and mathematical modelling literature [6, 48]. These findings support the inclusion of ivermectin for STH control in areas where this species is endemic.

We found a significant reduction in hookworm prevalence with ivermectin and albendazole, and no reduction with ivermectin alone, in agreement with an efficacy meta-analysis [6]. Surprisingly, reduced MDA coverage was associated with a greater hookworm prevalence reduction in our sensitivity analysis, potentially due to fewer MDA rounds in studies with ≥ 75% coverage and/or variations in unmeasured confounding, such as WASH access, socioeconomic status, or baseline infection intensity.

Although we observed a moderate reduction in *A. lumbricoides* prevalence with ivermectin alone, we did not detect a significant reduction with ivermectin and albendazole MDA. This was unexpected given that the efficacy of albendazole against *A. lumbricoides* infections is well documented [6]. We hypothesise that our finding may reflect low MDA coverage (with two of the five studies having less than 35% coverage [25, 36]) and/or the longer time period from the last round of MDA to the follow-up assessment in the albendazole and ivermectin studies (average 13.3 months across 5 studies) compared to ivermectin only studies (average 4.7 months across 3 studies). This could be especially impactful for *A. lumbricoides* due its high fecundity and egg survival rates, leading to increased environmental contamination and consequently rapid rates of reinfection and new infections after MDA [49–51].

Meta-regression findings showed that increased baseline prevalence predicted a reduction in MDA effectiveness against *T. trichiura* and *A. lumbricoides*, and more rounds of MDA predicted enhanced effectiveness against hookworm, in agreement with mathematical modelling [52].

The publication of the WHO’s 2010–2020 NTD roadmap and the signing of the 2012 London Declaration on NTDs represented a global commitment to treat 75% of children at risk of STH infections through school-based targeted PC programs in all endemic countries, largely relying on international health aid and pharmaceutical donations [53]. There was exceptional progress made towards this goal, where 69% of the 596 million at-risk school-aged children received a benzimidazole tablet by 2017 [54]. Notably, many countries continue to rely on such drug donations to maintain impact. However, these drugs are usually designated for specific risk populations and diseases, such as albendazole for STH control in school-aged children or ivermectin for onchocerciasis control, limiting their reach. Furthermore, there has been a strategic shift in the 2021–2030 roadmap, to move from reliance on international health aid to increased country ownership of control programs supported by domestic funding [53, 55]. Our findings provide evidence to support additional drug supply and funding mechanisms to procure ivermectin and albendazole to enable the implementation of community-wide STH control programs. This would be particularly impactful in areas endemic with *S. stercoralis* or *T. trichiura*, and where there is significant geographic overlap with other NTDs that rely on ivermectin, including onchocerciasis, lymphatic filariasis, and scabies.

There were limitations to our study. There were a small number of studies, many quasi-experimental or observational in nature, highlighting a need for more methodologically rigorous studies evaluating the effectiveness of ivermectin-based MDA for STH control. Although we made use of the available evidence, we had insufficient data to draw reliable conclusions for *A. lumbricoides* and to conduct a direct comparison of drug regimens. There was significant heterogeneity in prevalence reduction for all syntheses, likely due to programmatic and population variation between studies, including socioeconomic and environmental conditions and WASH access. There was evidence of potential publication bias, possibly leading to an overestimation of the true effectiveness of ivermectin PC. Finally, there was an insufficient number of studies reporting infection intensity to allow pooling of results. Measuring changes in prevalence alone fails to capture the full benefits of PC given that higher intensity infections are an important indicator of clinical morbidity [56]. Future studies should evaluate effectiveness using both infection prevalence and intensity outcomes, aligning with WHO targets [55].

## Conclusions

Overall, our study underscores the key role of ivermectin-based MDA in addressing the shortcomings of the current global guidelines for STH control. Based on the findings of our study, revising international guidelines to include ivermectin in STH control programs is a promising option for the integrated control and eventual elimination of STHs and other NTDs as public health problems. Achieving this requires a well-coordinated effort that leverages international health aid and mobilises domestic resources effectively.

### Supplementary Information

**Additional file 1: Appendix S1.** Search strategy (PubMed). **Table S1.** List of studies excluded at full-text screening stage. **Table S2.** Quality assessment of studies included in meta-analysis. **Figure S1.** Doi plots and LFK index for ivermectin preventive chemotherapy studies for *A. lumbricoides* (a), *T. trichiura* (b), Hookworm (c), and *S. stercoralis* (d). **Figure S2.** Doi plots and LFK index for ivermectin and albendazole preventive chemotherapy studies for *Ascaris lumbricoides* (a), *Trichuris trichiura* (b), Hookworm (c), and for all studies for *Strongyloides stercoralis* (d). **Table S3.** Pooled prevalence reduction in sensitivity analyses.

## Data Availability

The data supporting the conclusions of this article are included in the article and its additional file. The raw data used and/or analysed during the current study are available from the corresponding author upon reasonable request.

## Abbreviations

LFK index: Luis Furuya-Kanamori index
MDA: Mass drug administration
NTD: Neglected tropical disease
PC: Preventive chemotherapy
STH: Soil-transmitted helminth
WASH: Water, sanitation and hygiene
WHO: World Health Organization
*CI*: Confidence interval
*OR*: Odds ratio
