# Supplementary material for: Effectiveness of ivermectin mass drug administration in the control of soil-transmitted helminth infections in endemic populations: a systematic review and meta-analysis
Source: Infect Dis Poverty. 2024 Feb 18;13:16. doi: 10.1186/s40249-024-01185-5 (PMC10874526; doi:10.1186/s40249-024-01185-5)
Supplement: Supplementary file 1 — Additional file 1: Appendix S1. Search strategy (PubMed). Table S1. List of studies excluded at full-text screening stage. Table S2. Quality assessment of studies included in meta-analysis. Figure S1. Doi plots and LFK index for ivermectin preventive chemotherapy studies for A. lumbricoides (a), T. trichiura (b), Hookworm (c), and S. stercoralis (d). Figure S2. Doi plots and LFK index for ivermectin and albendazole preventive chemotherapy studies for Ascaris lumbricoides (a), Trichuris trichiura (b), Hookworm (c), and for all studies for Strongyloides stercoralis (d). Table S3. Pooled prevalence reduction in sensitivity analyses. [file 40249_2024_1185_MOESM1_ESM.docx]

**Appendix S1: Search strategy (PubMed)**

1. Ivermectin [text word] or Ivermectin [MeSH term]
2. Helminth [text word] or Helminth [MeSH term]
3. Helminths [text word] or Helminths [MeSH term]
4. Soil-transmitted helminth [text word] or Soil-transmitted helminth [MeSH term]
5. Soil-transmitted helminths [text word] or Soil-transmitted helminths [MeSH term]
6. STH [text word]
7. Nematode [text word]
8. Geohelminth [text word]
9. Ascaris [text word] or ascaris [MeSH term]
10. Necator [text word] or necator [MeSH term]
11. Ancylostoma [text word] or ancylostoma [MeSH term]
12. Trichuris [text word] or trichuris [MeSH term]
13. Strongyloides [text word] or Strongyloides [MeSH term]
14. Roundworm [text word]
15. Whipworm [text word]
16. Helminthiasis [MeSH term]
17. Ascariasis [MeSH term]
18. Trichuriasis [MeSH term]
19. Ascaris lumbricoides [text word]
20. Trichuris trichiura [text word]
21. Necator americanus [text word]
22. Strongyloides stercoralis [text word]
23. Ancylostoma ceylanicum [text word]
24. Ancylostoma duodenale [text word]
25. **(2 or 3 or 4 or 5 or 6 or 7 or 8 or 9 or 10 or 11 or 12 or 13 or 14 or 15 or 16 or 17 or 18 or 19 or 20 or 21 or 22 or 23 or 24)**
26. Preventive chemotherapy [text word] or preventive chemotherapy [MeSH term]
27. Mass drug administration [text word] or mass drug administration [MeSH term]
28. Mass administration [text word] or mass administration [MeSH term]
29. Mass treatment [text word]
30. Population [text word]
31. community [text word]
32. communities [text word]
33. school [text word]
34. schools [text word]
35. village [text word]
36. villages [text word]
37. program [text word]
38. programme [text word]
39. **(26 or 27 or 28 or 29 or 30 or 31 or 32 or 33 or 34 or 35 or 36 or 37 or 38)**
40. **(1 and 25 and 39)**

| **Table S1. List of studies excluded at full-text screening stage.** | | |
| --- | --- | --- |
| **Author & Year** | **Reference** | **Reason for exclusion** |
| Adriko et al 2018 | PLoS Negl Trop Dis 12 (7): e0006520 | Did not investigate ivermectin preventive chemotherapy |
| Ali et al 2015 | 9th European Congress on Tropical Medicine and International Health. Basel Switzerland. (Conference abstract) | Prevalence of STH infection before and after chemotherapy was not provided |
| Barda et al 2017 | American Journal of Tropical Medicine and Hygiene 97 (3): 681-683 | Analysis and/or outcomes are only reported for positive cases |
| Behnke et al 1994 | Journal of Helminthology 68 (3): 187-195 | Randomization occurred at individual level |
| De Souza et al 2018 | 68th Annual Meeting of the American Society of Tropical Medicine and Hygiene. National Harbor USA. (Conference abstract) | Prevalence of STH infection before and after chemotherapy was not provided |
| Dembele et al 2012 | PLoS Neglected Tropical Diseases 6 (3): e1574 | Prevalence of STH infection before and after chemotherapy was not provided |
| Dembele et al 2018 | 68th Annual Meeting of the American Society of Tropical Medicine and Hygiene. National Harbor USA. (Conference abstract) | Duplicate data (from Dembele 2012) |
| Djeunga et al 2016 | 66th Annual Meeting of the American Society of Tropical Medicine and Hygiene. Baltimore USA. (Conference abstract) | Prevalence of STH infection before and after chemotherapy was not provided |
| Djeunga et al 2018 | 68th Annual Meeting of the American Society of Tropical Medicine and Hygiene. National Harbor USA. (Conference abstract) | Cross-sectional study |
| Djune-Yemeli et al 2020 | PLoS neglected tropical diseases 14 (11): e0008794 | Cross-sectional study |
| Dorkenoo et al 2013 | 62nd Annual Meeting of the American Society of Tropical Medicine and Hygiene. Washington USA. (Conference abstract) | Prevalence of STH infection before and after chemotherapy was not provided |
| Echazu et al 2014 | 63rd Annual Meeting of the American Society of Tropical Medicine and Hygiene. New Orleans USA. (Conference abstract) | Duplicate data (from Echazu 2017) |
| Eneanya et al 2021 | American Journal of Tropical Medicine and Hygiene 105 (5): 139-139 | Duplicate data (from Eneanya 2021) |
| Fossuo et al 2016 | 66th Annual Meeting of the American Society of Tropical Medicine and Hygiene. Baltimore USA. (Conference abstract) | Prevalence of STH infection before and after chemotherapy was not provided |
| Gankpala 2015 | 65th Annual Meeting of the American Society of Tropical Medicine and Hygiene. Atlanta USA. (Conference abstract) | Conference abstract (full-text not available) |
| Gann et al 1994 | J Infect Dis 169 (5): 1076-9 | Randomization occurred at individual level |
| Gutman et al 2010 | Am J Trop Med Hyg 83 (3): 534-41 | Cross-sectional study |
| Hailu et al 2020 | Infect Dis (Auckl) 13 (1): 1178633720932540 | Chemotherapy only given to positive cases |
| Hamidu et al 2017 | 8th Forum of the European and Developing Countries Clinical Trials Partnership. Lusaka Zambia. (Conference abstract) | Did not investigate ivermectin preventive chemotherapy |
| Hardy et al 2020 | PLoS neglected tropical diseases 14 (3): e0008106 | Cross-sectional study |
| Hardy et al 2017 | 67th Annual Meeting of the American Society of Tropical Medicine and Hygiene. New Orleans USA. (Conference abstract) | Duplicate data (from Hardy 2020) |
| Hardy et al 2019 | 68th Annual Meeting of the American Society of Tropical Medicine and Hygiene. National Harbor USA. (Conference abstract) | Conference abstract (full-text not available) |
| Hays et al 2017 | PLoS Neglected Tropical Diseases 11 (7): e0005825 | Chemotherapy only given to positive cases |
| Heukelbach et al 2004 | Arzneimittelforschung-Drug Research 54 (7): 416-421 | Chemotherapy only given to positive cases |
| Hurlimann et al 2022 | The Lancet Infectious Diseases 22 (1): 123-135 | Randomization occurred at individual level |
| Kearns et al 2011 | 7th European Congress on Tropical Medicine and International Health. Barcelona Spain. (Conference abstract) | Duplicate data (from Kearns 2017) |
| Kearns et al 2017 | PLoS Neglected Tropical Diseases 11 (5): e0005607 | Chemotherapy only given to positive cases |
| Keller et al 2021 | PLoS Neglected Tropical Diseases 15 (6): e0009561 | Chemotherapy only given to positive cases |
| Khieu et al 2013 | PLoS Neglected Tropical Diseases 7 (2): e2035 | Chemotherapy only given to positive cases |
| Khieu et al 2014 | Parasitology International 63 (5): 708-712 | Chemotherapy only given to positive cases |
| Khieu et al 2011 | 60th Annual Meeting of the American Society of Tropical Medicine and Hygiene. Philadelphia USA. (Conference abstract) | Duplicate data (from Khieu 2013) |
| Knopp et al 2009 | 6th European Congress on Tropical Medicine and International Health and 1st Mediterranean Conference on Migration and Travel Health. Verona Italy. (Conference abstract) | Duplicate data (from Knopp 2009) |
| Knopp et al 2010 | Clinical Infectious Diseases 51 (12): 1420-1428 | Chemotherapy only given to positive cases |
| Kositz et al 2022 | International Journal of Infectious Diseases 125 (1): 258-264 | Prevalence of STH infection before and after chemotherapy was not provided |
| Krolewiecki et al 2011 | 60th Annual Meeting of the American Society of Tropical Medicine and Hygiene. Philadelphia USA. (Conference abstract) | Prevalence of STH infection before and after chemotherapy was not provided |
| Krotneva et al 2015 | PLoS Negl Trop Dis 9 (9): e0004051 | Prevalence of STH infection before and after chemotherapy was not provided |
| Loukouri et al 2018 | 68th Annual Meeting of the American Society of Tropical Medicine and Hygiene. National Harbor USA. (Conference abstract) | Duplicate data (from Loukouri 2020) |
| Loukouri et al 2019 | Journal of Tropical Medicine 2019 (1): Article ID 7658594 | Prevalence of STH infection before and after chemotherapy was not provided |
| Maegga et at al 2016 | Tanzania Journal of Health Research 8 (2): 70-74 | Analysis and/or outcomes are only reported for positive cases |
| Matamoros et al 2021 | Clin Infect Dis 73 (7): 1203-1210 | Randomization occurred at individual level |
| Miller et al 2018 | Trop Med Infect Dis 3 (2): 48 | Analysis and/or outcomes are only reported for positive cases |
| Mohammed et al 2008 | PLoS Neglected Tropical Diseases 2 (1): e171 | Prevalence of STH infection before and after chemotherapy was not provided |
| Mohammed et al 2012 | Parasites and Vectors 5 (1): 299 | Prevalence of STH infection before and after chemotherapy was not provided |
| Moncayo et al 2008 | PLoS Negl Trop Dis 2 (9): e293 | Cross-sectional study |
| Moser et al 2017 | The Lancet Infectious Diseases 17 (11): 1162-1171 | Randomization occurred at individual level |
| Mupfasoni et al 2016 | PLoS Neglected Tropical Diseases 10 (12): e0005202 | Prevalence of STH infection before and after chemotherapy was not provided |
| Ndyomugyenyi et al 2008 | Am J Trop Med Hyg 79 (6): 856-63 | Randomization occurred at individual level |
| Oluwole et al 2017 | Parasite Epidemiology and Control 2 (2): 21-29 | Cross-sectional study |
| Paltridge et al 2019 | The American journal of tropical medicine and hygiene. 2 (2): 339–345 | Chemotherapy only given to positive cases |
| Parker et al 2011 | Health Research Policy and Systems 9 (1): 3 | Qualitative research |
| Pion et al 2017 | 67th Annual Meeting of the American Society of Tropical Medicine and Hygiene. New Orleans USA. (Conference abstract) | Did not investigate ivermectin preventive chemotherapy |
| Ramos et al 2017 | 67th Annual Meeting of the American Society of Tropical Medicine and Hygiene. New Orleans USA. (Conference abstract) | Analysis and/or outcomes are only reported for positive cases |
| Ranque et al 2001 | Annals of Tropical Medicine and Parasitology 95 (4): 389-393 | Chemotherapy only given to positive cases |
| Rowbotton et al 2012 | Boletin De Malariologia Y Salud Ambiental 52 (2): 195-209 | Chemotherapy only given to positive cases |
| Shimogawara et al 2013 | European Journal of Microbiology and Immunology 3 (4): 258-266 | Chemotherapy only given to positive cases |
| Speich et al 2015 | Lancet Infect Dis 15 (3): 277-84 | Randomization occurred at individual level |
| Speich et al 2016 | Parasites and Vectors 9 (1): 123 | Randomization occurred at individual level |
| Supali et al 2017 | American Journal of Tropical Medicine and Hygiene 97 (5): 614-614 | Analysis and/or outcomes are only reported for positive cases |
| Taticheff et al 1994 | Ethiopian Medical Journal 32 (1): 7-15 | Chemotherapy only given to positive cases |
| Toma et al 2000 | The Southeast Asian journal of tropical medicine and public health 31 (2): 383-387 | Chemotherapy only given to positive cases |
| Vargas et al 2015 | 65th Annual Meeting of the American Society of Tropical Medicine and Hygiene. Atlanta USA. (Conference abstract) | Duplicate data (from Vargas 2017) |
| Welsche et al 2022 | The Lancet Infectious Diseases 28 (3): 331-340 | Randomization occurred at individual level |
| Whitworth et al 1991 | Trans R Soc Trop Med Hyg 85 (2): 232-4 | Randomization occurred at individual level |
| Whitworth et al 1988 | The Lancet 332 (8602): 97-8 | Duplicate data (from Whitworth 1991) |

| **Table S2. Quality assessment of studies included in meta-analysis.** | | | | | | | | | |
| --- | --- | --- | --- | --- | --- | --- | --- | --- | --- |
| **Author & Year** | **External validity** | | | | **Internal validity** | | | | |
|  | The target population was clearly described and was a close representation of the general population* in relation to relevant variables, e.g. age and sex. | The sampling frame was clearly described and was a true or close representation of the target population. | Some form of random selection was used to select the sample, or a census was undertaken. | Response rate was ≥75%, or analysis showed no significant difference in relevant demographic characteristics between responders & nonresponders. | The STH detection method was clearly described and was reliable and valid. | Same method of STH diagnosis was used for all subjects in the study. | Subjects were selected or recruited from the same or similar populations at both time points, or the same cohort was followed longitudinally. | The sampling method was well described, and the same method was used at both time points, or the same cohort was followed longitudinally. | The distribution strategy of deworming medication was clearly described and delivered to at least 75% of the target population. |
| Anselmi et al 2015 | No | No | No | No | Yes | No | No | No | Yes |
| Bah et al 2019 | Yes | Yes | Yes | No | Yes | Yes | Yes | Yes | Yes |
| Echazu et al 2017 | Yes | Yes | Yes | No | Yes | Yes | Yes | Yes | Yes |
| Eneanya et al 2021 | Yes | Yes | No | No | Yes | Yes | Yes | Yes | No |
| Eneanya et al 2022 | Yes | Yes | Yes | No | Yes | Yes | Yes | Yes | No |
| Heukelbach et al 2004 | Yes | No | Yes | Yes | Yes | Yes | Yes | Yes | Yes |
| Hurlimann et al 2018 | Yes | Yes | Yes | No | Yes | Yes | Yes | Yes | No |
| Knudson et al 2012 | No | No | No | No | Yes | Yes | No | No | Yes |
| Le et al 2023a | Yes | Yes | Yes | No | Yes | Yes | Yes | Yes | Yes |
| Le et al 2023b | No | Yes | No | No | Yes | Yes | Yes | Yes | Yes |
| Loukouri et al 2020 | Yes | Yes | Yes | No | Yes | Yes | Yes | Yes | No |
| Marks et al 2020 | Yes | Yes | Yes | No | Yes | Yes | Yes | Yes | Yes |
| Massa et al 2009 | Yes | Yes | Yes | Yes | Yes | Yes | Yes | Yes | Yes |
| Vargas et al 2017 | Yes | No | Yes | No | Yes | Yes | Yes | Yes | No |
| Ziem et al 2006b | Yes | Yes | Yes | No | Yes | Yes | Yes | Yes | No |
| *Population under study for STH prevalence (either school-aged children or whole community). | | | | | | | | | |

**
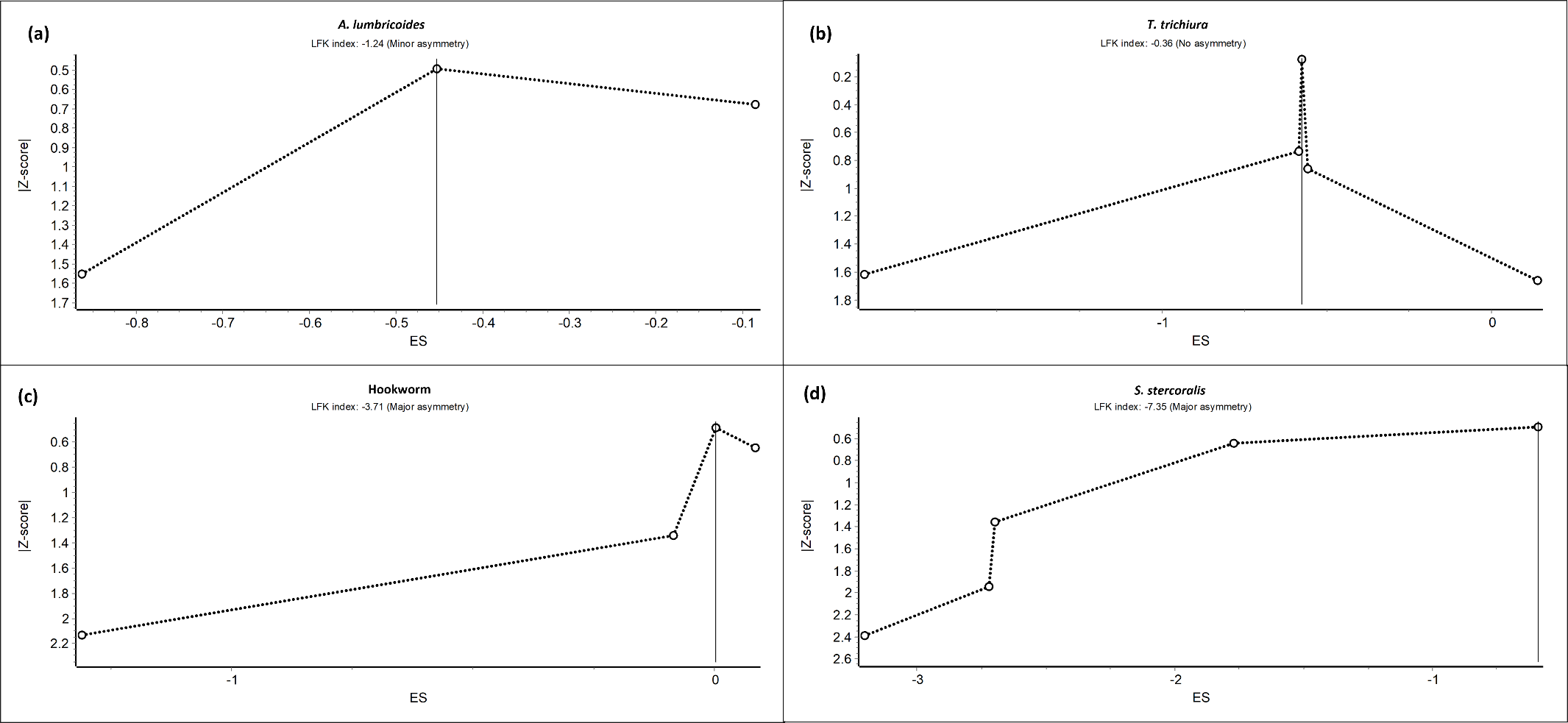
 Figure S1. Doi plots and LFK index for ivermectin preventive chemotherapy studies for *A. lumbricoides* (a), *T. trichiura* (b), Hookworm (c), and *S. stercoralis* (d).**

**Figure S2. Doi plots and LFK index for ivermectin and albendazole preventive chemotherapy studies for *Ascaris lumbricoides* (a), *Trichuris trichiura* (b), Hookworm (c), and for all studies for *Strongyloides stercoralis* (d).**
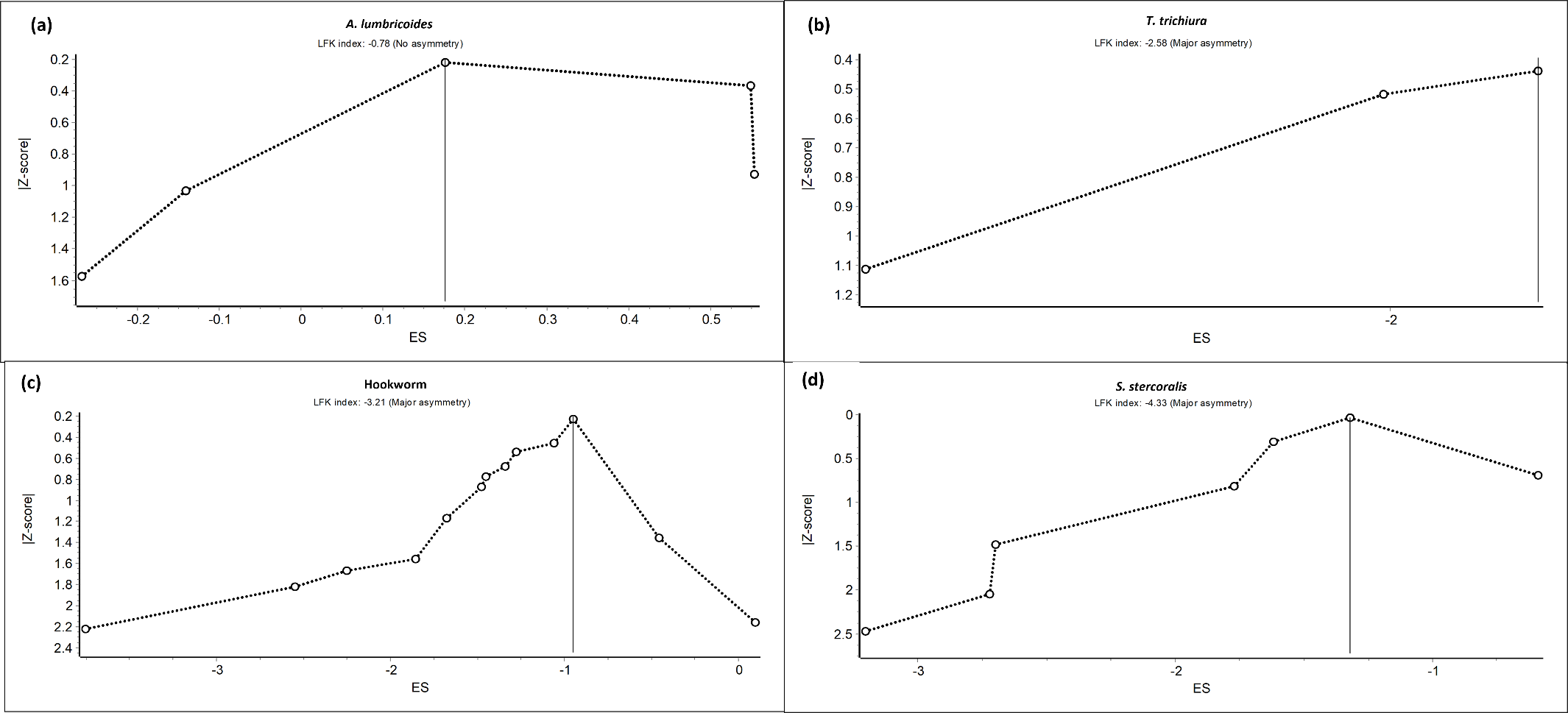


| **Table S3. Pooled prevalence reduction in sensitivity analyses.** | | | | |
| --- | --- | --- | --- | --- |
| **Restriction criteria** | ***N*** | **Ivermectin with or without albendazole**  **Pooled prevalence reduction (95% *CI*)** | | |
| ***S. stercoralis* studies** |  |  | | |
| Ivermectin and albendazole | 2 | 78.64% (55.70–89.70) | | |
| Ivermectin alone | 5 | 84.49% (54.96–94.66) | | |
| Follow-up time from last round: >6 months | 5 | 78.42% (53.95–89.89) | | |
| Follow-up time from last round: >6 months & ≤18 months | 4 | 77.34% (41.51–91.22) | | |
| Stool-based diagnostic methods | 5 | 85.93% (76.94–91.41) | | |
|  | **Ivermectin alone** | | **Ivermectin and albendazole** | |
|  | ***N*** | **Pooled prevalence reduction (95% *CI*)** | **N** | **Pooled prevalence**  **reduction (95% *CI*)** |
| ***T. trichiura* studies** | | | | |
| Follow-up time from last round: >6 months | 3 | 43.70% (32.68–52.92) | 0 | Insufficient data |
| Kato Katz diagnostic method | 0 | Insufficient data | 2 | 90.31% (66.78–97.17) |
| Number of preventive chemotherapy rounds: 1 | 3 | 43.70% (32.68–52.92) | 1 | Insufficient data |
| Number of preventive chemotherapy rounds: >1 | 2 | 58.66% (-205.57–94.41) | 2 | 90.31% (66.78–97.17) |
| **Hookworm studies** | | | | |
| Follow-up time from last round: >6 months | 3 | 29.15% (-8.33–53.66) | 10 | 81.45% (67.43–89.44) |
| Follow-up time from last round: >6 months & ≤18 months | 1 | Insufficient data | 8 | 75.51% (59.52–85.19) |
| Kato Katz diagnostic method | 0 | Insufficient data | 10 | 83.00% (72.34–89.55) |
| Number of preventive chemotherapy rounds: 1 | 3 | 29.15% (-8.33–53.66) | 4 | 47.14% (0.95–71.79) |
| Number of preventive chemotherapy rounds: >1 | 1 | Insufficient data | 9 | 85.70% (75.35–91.70) |
| Intervention coverage: <75% | 0 | Insufficient data | 7 | 88.95% (79.07–94.16) |
| Intervention coverage: ≥75% | 4 | NA | 4 | 48.80% (19.73–67.34) |
| ***A. lumbricoides* studies** | | | | |
| Follow-up time from last round: >6 months | 1 | Insufficient data |  | NA |
| Kato Katz diagnostic method | 0 | Insufficient data | 3 | -17.57% (-74.24–20.67) |
| Number of preventive chemotherapy rounds: 1 | 1 | Insufficient data | 2 | -0.27% (-73.69–42.12) |
| Number of preventive chemotherapy rounds: >1 | 2 | 23.25% (-10.02–46.46) | 3 | -17.57% (-74.24–20.67) |
| NA, not applicable as restriction criteria applies to all studies included in the primary analysis. | | | | |
